# *C. difficile* 630Δerm Spo0A Regulates Sporulation, but Does Not Contribute to Toxin Production, by Direct High-Affinity Binding to Target DNA

**DOI:** 10.1371/journal.pone.0048608

**Published:** 2012-10-31

**Authors:** Katharina E. Rosenbusch, Dennis Bakker, Ed J. Kuijper, Wiep Klaas Smits

**Affiliations:** Department of Medical Microbiology, Leiden University Medical Center, Leiden, The Netherlands; Cornell University, United States of America

## Abstract

*Clostridium difficile* is a Gram positive, anaerobic bacterium that can form highly resistant endospores. The bacterium is the causative agent of *C. difficile* infection (CDI), for which the symptoms can range from a mild diarrhea to potentially fatal pseudomembranous colitis and toxic megacolon. Endospore formation in Firmicutes, including *C. difficile*, is governed by the key regulator for sporulation, Spo0A. In *Bacillus subtilis*, this transcription factor is also directly or indirectly involved in various other cellular processes. Here, we report that *C. difficile* Spo0A shows a high degree of similarity to the well characterized *B. subtilis* protein and recognizes a similar binding sequence. We find that the laboratory strain *C. difficile* 630Δerm contains an 18bp-duplication near the DNA-binding domain compared to its ancestral strain 630. *In vitro* binding assays using purified C-terminal DNA binding domain of the *C. difficile* Spo0A protein demonstrate direct binding to DNA upstream of *spo0A* and *sigH*, early sporulation genes and several other putative targets. *In vitro* binding assays suggest that the gene encoding the major clostridial toxin TcdB may be a direct target of Spo0A, but supernatant derived from a *spo0A* negative strain was no less toxic towards Vero cells than that obtained from a wild type strain, in contrast to previous reports. These results identify for the first time direct (putative) targets of the Spo0A protein in *C. difficile* and make a positive effect of Spo0A on production of the large clostridial toxins unlikely.

## Introduction

Sporulation is an adaptive strategy that enables bacteria to survive harsh environmental conditions for prolonged periods of time, and is an integral part of the transmission of sporulating pathogens and their tolerance and resistance towards antimicrobial compounds.

Spo0A is the key regulator for sporulation [1], [2]. Most of our knowledge about the protein is based on work in *Bacilli*. Spo0A is a response regulator that demonstrates phosphorylation dependent binding to DNA [3]–[5]. Phosphorylation occurs through the concerted action of several proteins that together form a so called phosphorelay [6]. The signaling cascade allows for the integration of environmental signals into the regulation of Spo0A dependent processes, including sporulation. The two functional domains, the N-terminal phosphorylation and dimerization domain (receiver domain), and the C-terminal DNA binding (effector) domain are separated by a hinge region that is relatively poorly conserved [7]. Phosphorylation is believed to result in a structural rearrangement that facilitates dimerization [8], [9], resulting in the disruption of transcription-inhibitory contacts between the receiver and effector domains. The isolated DNA binding domain can bind legitimate targets of the Spo0A protein due to the absence of the transcription inhibitory contacts, thereby bypassing the need for phosphorylation [10]. Extensive characterization of Spo0A targets has revealed a motif that represents a high affinity Spo0A binding site, the 0A box [10], [11]. The crystal structure of the DNA binding domain confirms specific and non-specific contacts between the protein and the consensus sequence [12], [13]. It is noteworthy that Spo0A regulates many other processes than sporulation, such as competence for genetic transformation, DNA replication, and biofilm formation in *B. subtilis* [14]–[16], virulence factors and stress responses in for instance *B. anthracis* and *B. thuringiensis* [17]–[21], and solvent production in *Clostridium acetobutylicum* [22], [23].

*C. difficile* is a Gram positive, anaerobic bacterium that is the causative agent of *C. difficile* infection (CDI) (for recent reviews see [24], [25]). Though many people are asymptomatically colonized by *C. difficile*, the bacterium can cause serious health problems, such as pseudomembranous colitis and toxic megacolon, under the influence of risk factors such as age and antibiotic use. As a result, CDI was long regarded a nosocomial infection. Recently, however, an increase in the cases of community acquired CDI can be observed [26]. Outbreaks of CDI have been linked to so called hypervirulent strains, such as PCR ribotypes 027 (BI/NAP1) and 078 [27], [28]. Its main virulence factors are the major clostridial toxins A and B [29], [30]. In addition, certain strains of *C. difficile*, including ribotypes 027 and 078, additionally encode a binary toxin [31], [32]. *C. difficile* is transmitted via the fecal-oral route. It is believed that spores are crucial to successfully infect new hosts, as they are able to withstand the harsh environment of the stomach, and survive antibiotic treatments that alter the endogenous flora, after which *C. difficile* can overgrow [24], [25].

There is limited knowledge about the regulation of sporulation in *C. difficile*. It has been reported that *spo0A,* as expected, is required for the formation of spores [33] and the gene is required for persistence and transmission in mice [34]. Though the pathways downstream of Spo0A seem to a large extent conserved between *B. subtilis* and *Clostridia*, this is less so for the pathways leading to activation of Spo0A [2]. It has been suggested that the orphan histidine kinase CD2492 is involved in the activation of Spo0A [35]. Similarly, it was reported that multiple orphan histidine kinases can phosphorylate Spo0A in *C. acetobutylicum* [36]. Recently, it was reported that *spo0A* can be transcribed from a SigH-dependent promoter [37]. It is unknown which genes are regulated by direct binding of Spo0A to their upstream regions.

Here, we establish an *in vitro* binding assay for *C. difficile* Spo0A and demonstrate for the first time direct binding of this transcription factor to DNA upstream of several putative target genes.

## Methods

### Bacterial Strains and Media

*Escherichia coli* strains were routinely grown in Luria-Bertani broth or plates, supplemented with appropriate antibiotics. Chloramphenicol was used at a final concentration of 20 µg/mL for agar plates and 10 µg/mL for liquid cultures. Ampicillin was used at a final concentration of 100 µg/mL. Kanamycin was used at a final concentration of 20 µg/mL. Cloning was carried out using *E. coli* DH5α, overexpression was performed in *E. coli* Rosetta(DE3) pLysS (Novagen). *C. difficile* strains were grown in a glucose-free trypton-yeast based medium (TTY; 3% w/v bacto-trypton (BD), 2% yeast extract (Fluka), 0.1% w/v thioglycollate (Sigma) pH 7.4), supplemented with 20 µg/mL of lincomycin when appropriate, or on CLO or TSS plates (Biomerieux).

### Plasmid Construction

All plasmids are listed in Table 1. Primers (obtained from Sigma Aldrich) are listed in Text S1 and specific cycling conditions are available on request. Unless noted otherwise, PCR reactions were carried out using Pfu polymerase (Fermentas) according to the instructions of the manufacturer.

**Table 1:** Plasmids used in this study.

Name	Description*	Reference
pMF14	pET21b-*spo0A*-DBD-6xHis (Bsu)	[10]
pWKS1245	pET21b-*spo0A*-DBD-6xHis (Cdi)	This study
pWKS1251	pET21b-*spo0A*-6xHis (Cdi)	This study
pWKS1303	pCR2.1TOPO-P*abrB* (Bsu)	This study
pWKS1304	pCR2.1TOPO-P*abrB* (C4T)	This study
pWKS1305	pCR2.1TOPO-P*abrB* (C4T/G5T)	This study
pWKS1306	pCR2.1TOPO-P*abrB* (G2T)	This study
pWKS1307	pCR2.1TOPO-P*abrB* (G2T/C4T)	This study
pWKS1308	pCR2.1TOPO-P*abrB* (G5T)	This study
pWKS1309	pCR2.1TOPO-P*citG* (Bsu)	This study
pWKS1316	pCR2.1TOPO-P*spo0A* (Cdi)	This study
pWKS1317	pCR2.1TOPO-P*spoIIAA* (Cdi)	This study
pWKS1318	pCR2.1TOPO-P*spoVG* (Cdi)	This study
pWKS1322	pCR2.1TOPO-P*sigH* (Cdi)	This study
pWKS1323	pCR2.1TOPO-P*spoIIGA* (Cdi)	This study
pWKS1324	pCR2.1TOPO-P*spoIIE* (Cdi)	This study
pWKS1326	pCR2.1TOPO-P*tcdB* (Cdi)	This study
pWKS1329	pCR2.1TOPO-P*tcdA* (Cdi)	This study
pWKS1332	pCR2.1TOPO-P*lplA* (Cdi)	This study
pWKS1340	pCR2.1TOPO-P*tcdR* (Cdi)	This study
pWKS1341	pCR2.1TOPO-P*ssuA* (Cdi)	This study

* Bsu  =  DNA derived from *B. subtilis* JH642. Cdi  =  DNA derived from *C. difficile* 630Δerm.

Plasmid pWKS1251, for the overproduction of Spo0A-DBD carrying a C-terminal 6×His-tag, was constructed as follows. A sequence corresponding to the DNA binding domain of Spo0A was amplified using primers oWKS-1123a and oWKS-1124 using chromosomal DNA from *C. difficile* strain 630Δerm as a template. The resulting fragment was cloned into pCR2.1-TOPO (Invitrogen), yielding pWKS1247. This plasmid was digested with NdeI and XhoI, separated on a 1% agarose/0.5× TAE (20 mM Tris Acetate, 0.5 mM EDTA) gel, the fragment corresponding to the DNA binding domain was recovered by gel-isolation (using a GeneJET Gel Extraction kit, Fermentas) and cloned into similarly digested pMF14 [10] that had been gel-isolated in the same manner. The construct was verified by PCR, restriction analyses and DNA sequencing using primers oWKS-135 and oWKS-136 (see below).

Plasmid pWKS1245, for the production of full length Spo0A carrying a C-terminal 6xHis-tag, was constructed in a similar manner using chromosomal DNA from *C. difficile* 630Δerm as a template, but using the PCR product of primers oWKS-1122 and oWKS-1123a.

Plasmids used as PCR templates for generating EMSA probes were constructed by cloning the PCR products into pCR2.1-TOPO. The inserts, and in the case of the mutated P*abrB* promoters the presence of the desired point mutations in the consensus 0A box, were verified by DNA sequencing using primers oWKS-24 and oWKS-25 (see below).

### DNA Sequencing

Sequence grade plasmids were isolated using a Nucleospin Plasmid QuickPure kit (Macherey Nagel) according to the manufacturer’s instructions, except that two lysis reactions were combined onto a single filter and eluted with 65°C prewarmed AE buffer. All constructs were sequenced using BigDye Terminator chemistry (Invitrogen) on an ABI3130 sequencer (Perkin Elmer), according to the instructions of the manufacturers. In short, ∼200 ng of plasmid was mixed with 3.2 pmol of primer, 1 µL Terminator Ready Reaction Mix (Invitrogen) in a final volume of 20 µL. After thermocycling, DNA was precipitated and washed with 65% isopropanol, and dissolved in 12 µL HiDi formamid (Invitrogen) at 96°C for 2 mins and stored in the dark at 4°C until the sequencing run. Sequence analyses were performed in CloneManager Professional Suite 7 (SciEd) and Geneious version 5.6.2 (Biomatters Ltd).

### Protein Purification

Plasmids pWKS1245 and pWKS1251 were transformed into *E. coli* Rosetta(DE3) pLysS (Novagen). Transformants were used to inoculate 25 mL of LB with appropriate antibiotics. After overnight incubation, the cells were 1∶100 diluted in 500 mL fresh medium containing appropriate antibiotics. Protein production was induced with 1 mM IPTG at an OD600 of 0.7 and growth was continued for another three hours before harvesting. Cells were washed with ice cold PBS and stored at −80°C for later use.

Purification of the proteins was essentially done as described [10]. In short, cells were disrupted in 4 mL lysis buffer (2 mM PMSF, 10 mM imidazole, 5 mM beta-mercaptoethanol, 300 mM NaCl, 50 mM NaH_2_PO_4_, pH 7.9). Cleared cell lysates we incubated with 2 mL pre-equilibrated 50% TALON slurry (Clontech) in a final volume of 15 mL lysis buffer for 1 hr. The resin was allowed to settle on a Poly-Prep column (BioRad) and washed with 2 mL wash buffer (20 mM imidazole, 300 mM NaCl, 50 mM NaH_2_PO_4_, pH 7.9). The protein was stepwise eluted in 1 mL fractions after applying 2 mL elution buffer to the column (identical to wash buffer but with 50, 100, 250 or 500 mM imidazole). The whole procedure was carried out at 4°C. Fractions were assayed for purity and yield and suitable fractions were dialysed against 2× 1L dialysis buffer (50 mM Tris-HCl pH 8, 1 mM EDTA, 0.5 mM DTT) using Slide-A-Lyzer cassettes with a molecular weight cut-off of 3.5 kDa (Pierce).

Proteins were stored at −80°C in storage buffer (identical to dialysis buffer but containing 20% glycerol). Protein concentrations were determined using Bradford reagent (BioRad), according to the manufacturer’s instructions.

### Electrophoretic Mobility Shift Assays

DNA fragments for use in EMSA experiment were generated by PCR using GoTaq polymerase (Promega) and chromosomal DNA from *B. subtilis* JH642 (Bacillus Genetic Stock Center 1A96; http://www.bgsc.org), plasmids listed in Table 1, or chromosomal DNA from *C. difficile* 630Δerm [38] as a template. Primers and specific cycling conditions for generation of the EMSA probes are listed in Text S1. DNA fragments of the expected size were isolated from a 1×TAE/8% native polyacrylamide gel using diffusion buffer (0.5 M ammonium acetate, 10 mM magnesium acetate, 1 mM EDTA pH 8, 0.1% SDS) and a QIAExII kit (Qiagen), according to the manufacturer’s instructions. Recovered DNA was end-labeled with 32P-γ-ATP using FR buffer and T4 kinase (Invitrogen) according to the instructions of the manufacturer. Specific activity was determined on a LS6000 scintillation counter (Beckman).

EMSA conditions were based on previous studies [10]. In short, binding reactions were carried out in binding buffer (10 mM Tris-HCl pH 7.6, 1 mM EDTA, 50 mM NaCl, 1 mM DTT, 5% glycerol) in the presence of 200 µg/mL bovine serum albumin (NEB) and 200 cpm/µL radiolabeled DNA fragment. Reactions were incubated for 20 minutes at 30°C prior to loading on a 1×TAE/8% non-denaturing polyacrylamide gel that was prerun for 20 minutes at 50 V in 1× TAE buffer. Electrophoresis was carried out for 120 min at 85 V. After vacuum drying the gels onto filter paper, they were imaged after overnight exposure on Phosphorimager screens on a Typhoon instrument (GE Healthcare).

### Cytotoxicity Assay

The toxic effects of *C. difficile* culture supernatants on Vero cells (a kind gift of Eric Snijder [39]) were determined as follows. Supernatant from a bacterial culture was harvested by centrifuging cells for 3 minutes at 14000× g and filtered on a 0.45 µM cellulose acetate filter using a syringe. Supernatants were 2-fold serially diluted in cell culture medium (Dulbecco modified Eagle medium (Lonza) supplemented with 100 µg/mL penicillin, 100 U/mL streptomycin, 10% fetal calf serum), before applying them to a monolayer of Vero cells, and incubation was continued for another hour. As a positive control, 50 µL 1∶10 diluted purified toxin (Techlab) was added to the cells. To determine if observed cytotoxic effects were specific for the large clostridial toxins, commercially available anti-toxin against TcdA and TcdB (Techlab) was added to 10-fold diluted bacterial supernatant for 60 min prior to incubation on the Vero cells. Toxin end-point titres were defined as the lowest dilution at which no cytopathological effects (cell rounding) were observed.

Statistical significance was evaluated with an independent sample t-test.

### Generation of Antibodies Against Spo0A and Immunoblot Detection

Immunization of mice with full length *C. difficile* Spo0A-6xHis was kindly performed at the Welcome Trust Sanger Institute (Hinxton, UK). Cells from 1 mL of *C. difficile* culture were collected by centrifugation for 1 min at 14000 rpm in a table top centrifuge and resuspended in 200 µL resuspension buffer (10 mM Tris HCl pH 8, 10 mM EDTA, 0.5 mg/mL lysozyme, 1 mM Pefabloc SC (Roche)). After incubation for 30 mins at 37°C, 50 µL of 5× SDS sample buffer (0.1 M DTT, 2% SDS, 50 mM Tris HCl pH 6.8, 10% glycerol, 0.0025% BPB) was added, and samples were heated to 96°C for 5 mins. Total cell lysates (amounts corrected for OD_600_) were separated on a 12% SDS-PAGE gel prior to semi-dry blotting for 1 h at 10 V to a polyvinylidene fluoride (PVDF) membrane. Membranes were blocked in PBST buffer (phosphate buffered saline with 0.1% v/v Tween-20) containing 5% membrane blocking reagent (Amersham Biosciences). To visualize Spo0A protein cleared polyclonal serum from a single mouse at a 1∶3000 dilution was used, followed by either a goat-anti-mouse HRP-conjugated secondary antibody followed by ECL+ detection (Amersham Bioscience), or a goat-anti-mouse-biotin-conjugated secondary antibody (Dako) followed by a tertiary mouse-anti-biotin Cy3-conjugated antibody (Jackson). Detection was done using on a Typhoon instrument (GE Healthcare). Background corrected peak volumes were quantified using ImageQuant TL (Amersham Biosciences).

### Bioinformatics and Software

Alignments of *B. subtilis* and *C. difficile spo0A* were made using ClustalW2 (http://www.ebi.ac.uk/Tools/msa/clustalw2/) on the basis of the published genome sequences, Genbank accession numbers AL009126 and AM180355, respectively, and the 630Δerm *spo0A* sequence as determined in this study. The sequence for *spo0A* of *C. difficile* strain 630Δerm was deposited in Genbank (accession no JX050222). Consensus Spo0A boxes were identified using a Single string Search command in Genome2D [40], allowing 0 mismatches. The box positions were linked to up- and downstream genes using the “Add nearest gene to List of DNA Motifs” feature and Microsoft Excel. The results were manually inspected for those boxes within 500 bp upstream of a gene on the same strand. Figures for publication were prepared using ImageQuant TL (Amersham Biosciences), Adobe Photoshop CS3 (Adobe Systems Inc) and Corel Graphics Suite X5 (Corel Corporation).

## Results

### Purification of Spo0A and its DNA Binding Domain

**Figure 1:**
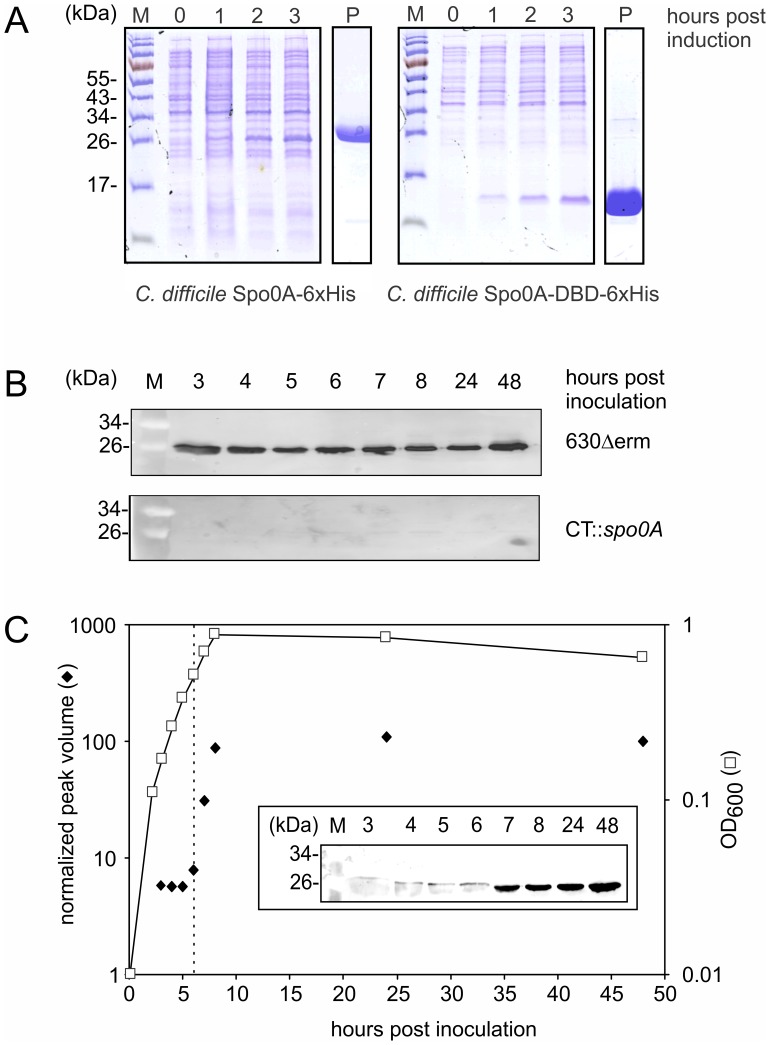
Purification and detection of *C. difficile* Spo0A. A. Heterologous overproduction of Spo0A-6xHis and Spo0A-DBD-6xHis in *E. coli* Rosetta(DE3) pLysS. M  =  molecular weight marker, numbers indicate hours after induction with 1 mM IPTG. P  =  metal affinity purified protein. Lysates were separated on a 12% SDS-PAGE. **B** *and* **C.** Immunoblot detection of Spo0A in total cell lysates of a *C. difficile spo0A* mutant (CT::spo0A) and a wild type strain (630Δerm). Times indicated range from early exponential (3 h post inoculation) to late stationary growth phase (48 h post inoculation). Sample volumes were corrected for OD600 to ensure loading of similar amounts of total cell lysate in each lane. For details see Materials and Methods. M  =  molecular weight marker. **B.** ECL+ detection. **C.** Fluorescent detection. Y-axes show peak volumes normalized to values at 48 hours post inoculation (closed diamonds; left axis) and optical density readings at 600 nM (open squares; right axis). Inset shows the blot on which the curve is based. Vertical dashed line indicates the moment Spo0A levels increase sharply (6 hours post inoculation).

In order to characterize *C. difficile* Spo0A, the full length protein and its DNA binding domain (DBD) were expressed as a C-terminally 6×His-tagged protein in the heterologous host *Escherichia coli* (Fig. 1A) and purified to near homogeneity using metal affinity chromatography (Fig. 1A; lanes P). Full length protein was used to raise antibodies to detect Spo0A in total lysates of *C. difficile* strains, and the purified DNA binding domain was used in subsequent *in vitro* binding assays (see below).

### Spo0A is Expressed throughout Growth

We determined the expression of *C. difficile* Spo0A throughout growth. We found that the protein is present in lysates from exponential to stationary growth phase cells. We performed immunoblotting using polyclonal antibodies against *C. difficile* Spo0A on total lysates of wild type and *spo0A* mutant cells grown in a trypton-yeast based medium (TTY). We found a clear signal of the size expected for full length Spo0A (∼31 kDa) as early as 3 hours post inoculation (exponential growth phase), through transition phase (8 h) as well as 24 and 48 hours post inoculation (stationary growth phase) (Figure 1B; 630Δerm). The signals were specific for *C. difficile* Spo0A as they were absent from lysates from the *C. difficile spo0A* mutant (Fig. 1B, CT::*spo0A*). We obtained similar results in other media, such as the commonly used supplemented brain heart infusion broth (BHIS; data not shown).

To determine relative levels of Spo0A throughout growth, we performed an immunoblot experiment using fluorescent antibodies, which gives more quantitative information compared to the use of horseradish peroxidase conjugated antibodies in our hands. We found that the levels of Spo0A increases approximately 20-fold from 6 hours post inoculation and remains at similar levels from 8 to 48 hours post inoculation (Figure 1C).

Though it should be noted that the Western blots do not provide information on the phosphorylation state of the protein, we conclude that the protein in active or inactive form is present throughout growth and is more abundant in stationary growth phase.

### Spo0A of *C. difficile* Strain 630Δerm Contains a 6-aminoacid Duplication

**Figure 2:**
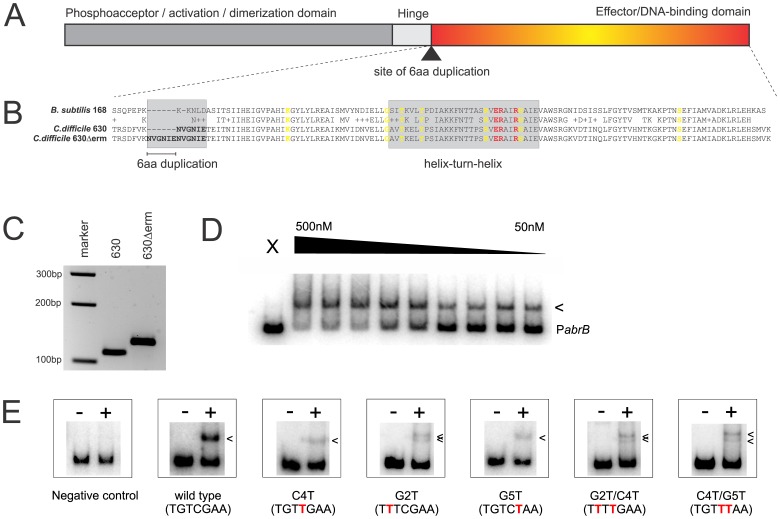
A Spo0A box is important for high affinity binding by *C. difficile* Spo0A. A. Domain organization of Spo0A. The site of the duplication in strain 630Δerm identified in this study is indicated by an arrow. **B.** Sequence alignment of the C-terminal regions of the Spo0A proteins of *B. subtilis* 168 and *C. difficile* strains 630 and 630Δerm. Residues identified in structural studies using *Bacillus* Spo0A as involved in backbone interactions are indicated in yellow, residues forming base-specific contacts are indicated in red [13]. The region of the 6aa duplication and the helix-turn-helix motifs are boxed in gray and the duplication in strain 630Δerm is underlined. **C.** PCR showing the presence of the duplication near the DNA binding domain in *C difficile* 630Δerm compared to 630. **D.** Electrophoretic mobility shift assay using purified *C. difficile* Spo0A-DBD-6xHis and a radiolabeled P*abrB* DNA fragment. X  =  no protein control, the triangle indicates 1.3-fold serial dilutions of protein to the indicated concentrations. The arrow indicates a DNA:protein complex. **E.** Electrophoretic mobility shift assays without (−) or with (+) 150 nM Spo0A-DBD-6xHis added to radiolabeled P*abrB* fragments carrying mutations in the consensus Spo0A box (in red). Arrows indicate DNA:protein complexes. The negative control is P*citG* from *B. subtilis*.

BLAST homology searches readily identify a homolog of the well-characterized *B. subtilis* Spo0A protein in *C. difficile* 630 (CD1214) and previous work demonstrated that a *spo0A* mutant (an insertional inactivation of *cd1214*) – as expected – no longer forms spores [41]. *In silico* analyses suggest a similar secondary structure for both proteins (Fig. 2A), with a conserved dimerization and DNA binding domain, separated by a poorly conserved hinge region [7], [12].

We compared the sequence of CD1214 obtained from our lab strain 630Δerm [38] to that of the published *C. difficile* 630 genome [42]. Strain 630Δerm is a spontaneous erythromycin sensitive strain, which is commonly used in mutagenesis studies and was obtained by serial passaging of strain 630 [33], [38]. The 630Δerm *spo0A* sequence (Genbank accession no JX050222) was derived from the expression plasmids constructed for this study, and confirmed in a whole genome sequence of strain 630Δerm generated in our lab (data not shown). We found that 630Δerm *spo0A* contains an 18 base pair direct repeat, resulting in a 6 amino acid (NVGNIE) duplication compared to the published reference sequence. The duplication maps to a region of the protein with relatively low sequence conservation (hinge), flanking the highly conserved DNA binding domain (Fig. 2A and B). We verified the absence of this duplication in strain 630 by PCR (Fig. 2C) as well as sequencing from the chromosomal DNA of *C. difficile* 630 (data not shown), to rule out an error in the original genome sequence and to demonstrate that the difference in size of the PCR product was specific to the 18 bp insertion. In addition, we checked several other strains of PCR ribotypes 12 (to which 630 and 630Δerm belong) by PCR, but the duplication was found to be unique to 630Δerm among the isolates tested (data not shown).

### *C. difficile* Spo0A-DBD Shows Similar Specificity as *B. subtilis* Spo0A-DBD

Next, we examined the conservation of the DNA binding domain of Spo0A (Spo0A-DBD) between *B. subtilis* and *C. difficile.* In *B. subtilis* amino acid residues contacting the backbone of the DNA and interacting with specific residues of the Spo0A binding sequence have been defined [13]. We found that all these residues were conserved in the *C. difficile* protein sequence (Fig. 2B), indicating that the protein likely recognizes a similar motif.

DNA binding by full length Spo0A in *B. subtilis* requires phosphorylation dependent dimerization [8], [9]. However, it was shown that the isolated DBD is capable of binding to legitimate targets of the full length protein [10]. Analogously, we purified the *C. difficile* Spo0A-DBD for use in *in vitro* binding assays. As no direct targets for the *C. difficile* protein have been reported so far, we used the upstream region of the *abrB* gene (P*abrB*) of *B. subtilis*. P*abrB* is commonly used as a high-affinity control in binding assays with the *B. subtilis* Spo0A or Spo0A-DBD protein [43], [44]. It is noteworthy that we failed to identify a homolog of *abrB* in *C. difficile* using BLAST, indicating that potential indirect regulation by Spo0A cannot occur through *abrB* in *C. difficile* as it does in *B. subtilis*. We found that *C. difficile* Spo0A-DBD bound with high affinity to P*abrB* (Fig. 2D and E). We performed electrophoretic mobility shift assays (EMSAs) using radiolabeled P*abrB* and increasing amounts of purified *C. difficile* Spo0A-DBD that was purified using a C-terminal 6×His-tag. The addition of protein leads to a dose-dependent retardation of the DNA fragment with an apparent K_D_ of <50 nM. In the same range of protein concentrations, no binding was observed for a negative control (a DNA fragment of *B. subtilis citG* [*45*]) (Fig. 2E), suggesting that binding was specific for the *abrB* promoter region.

*B. subtilis* Spo0A recognizes a distinct sequence (0A box), that is characterized by a 7 bp core motif (TGTCGAA) [10], [11]. Structural studies have revealed that the protein makes specific contacts with the G at position 2 (G2), and the C at position 4 (C4) and 5 (G5) of this motif [13]. We introduced G2A, C4A, G5A, G2A/C4A and C4A/G5A mutations in the perfect consensus core 0A-box present in P*abrB*. We found that the affinity of *C. difficile* Spo0A for these mutated P*abrB* fragments was highly reduced (Fig. 2E). We performed EMSAs using radiolabeled P*abrB* containing the mutated core sequence. For the single point mutations in the DNA, the affinity decreased ∼10-fold. There did not seem to be an additive effect of a second point mutation for the two combinations tested. None of the mutations abolished binding of *C. difficile* Spo0A completely, most likely as the result of binding of Spo0A to other (non-consensus) 0A boxes in the *abrB* promoter [44].

Taken together, we conclude that the guanine and cytosine residues in the core TGTCGAA motif of P*abrB* are important for specific binding of this fragment by *C. difficile* Spo0A-DBD.

### The Presence of a Consensus Spo0A Box has Predictive Value for Binding by *C. difficile* Spo0A-DBD

Above, we have established that the Spo0A-DBD of *C. difficile* is highly homologous to that of the *B. subtilis* Spo0A protein, and that the proteins recognize a similar consensus sequence (Fig. 2). Based on this information, we identified the several genes as putative direct targets of *C. difficile* Spo0A.

We queried the *C. difficile* 630 genome sequence for perfect matches to the core 0A box using Genome2D [40]. Such an analysis revealed the presence of 102 matching motifs, of which 45 were located within 500 bp of the initiating ATG of an open reading frame on the same strand (see Table S1). Our attention was drawn to *spo0A* and *sigH*, as these two genes were previously found to be regulated by Spo0A in *B. subtilis* and/or play important roles in sporulation [3], [46]–[48]. We found that *C. difficile* Spo0A bound to DNA sequences upstream of *spo0A* and *sigH*.

**Figure 3:**
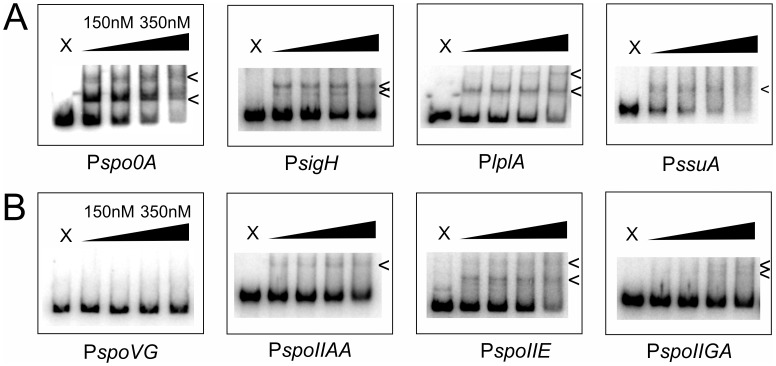
*C. difficile* Spo0A binds to predicted and expected target sequences. Electrophoretic mobility shift assay using purified *C. difficile* Spo0A-DBD-6xHis and a radiolabeled DNA fragments. X  =  no protein control, the triangle indicates 1.3-fold serial dilutions of protein to the indicated concentrations. The arrows indicate DNA:protein complexes. **A.** *In silico* predicted target sequences upstream of the genes encoding Spo0A (*spo0A*), σH (*sigH*), a lipoate ligase (*lplA*) and an aliphatic sulphonates ABC transporter (*ssuA*). **B.** Target sequences predicted on the basis of findings in other organisms: *spoIIAA*, *spoIIE* and *spoIIGA*. The DNA upstream of *spoVG* serves as a negative control.

We performed EMSAs with DNA encompassing 220–281 bp upstream of the initiating ATG codon of the *spo0A*, *sigH* and *spoVG* open reading frames. We found that the addition of Spo0A-DBD to the reactions caused retardation of the *spo0A* and *sigH* DNA fragments (Fig. 3A), but not of a *spoVG* fragment which did not contain a consensus 0A box (Fig. 3B). It should be noted that the affinity of Spo0A-DBD for the region upstream of *spo0A* was the highest we have observed so far for any *C. difficile* DNA. Moreover, the presence of multiple shifted species could indicate the presence of more than one strong binding site. These results establish that *spo0A* and *sigH* are likely legitimate targets of Spo0A in *C. difficile*, and confirm that *spoVG* is not, in line with results obtained in *B. subtilis* [10].

We were interested to see if Spo0A in *C. difficile* could potentially regulate genes that have no documented function in sporulation. Our *in silico* analysis identified several genes with no obvious link to sporulation that had a consensus 0A box within 100 bp upstream of their start codon. This positioning is similar to that observed for *spo0A* (−75) and *sigH* (−78). We confirmed *in vitro* binding of the *C. difficile* Spo0A-DBD to the promoter regions of *lplA* and *ssuA*.

We carried out EMSA experiments using probes that included the perfect consensus site and purified Spo0A-DBD protein. We observed binding of the protein to fragments upstream of the *lplA* gene (CD1654; box at −67) and the *ssuA* gene (CD1484; box at −82) (Fig. 3A). The *lplA* gene encodes a predicted lipoate-protein ligase, and *ssuA* is annotated as an aliphatic sulfonates ABC transporter; to our knowledge, neither of these have been directly implicated in sporulation or have found to be targets for Spo0A in other organisms.

Together our results establish the potential for binding of Spo0A to DNA upstream of *spo0A* and *sigH*, two genes that are important for sporulation, and indicate that Spo0A may have functions that go beyond the regulation of sporulation in *C. difficile*.

### *C. difficile* Spo0A-DBD Binds to Early Sporulation Promoters

It has been established that a *spo0A* mutant of *C. difficile* does not produce any spores, consistent with a crucial role in the sporulation pathway [33]. However, the *in silico* identification of upstream regions with a consensus Spo0A binding site did not point to any of the early sporulation genes (downstream of *spo0A* itself) as direct targets of Spo0A. This is likely the result of variations in the 0A-box in these promoters that were disregarded in the box search. In support of this, many well-characterized legitimate direct targets of *B. subtilis* Spo0A (such as *spoIIAA* and *spoIIE*) do not contain a 100% match to the core motif, but rather one or more near-consensus boxes [5], [49]. We found that Spo0A-DBD of *C. difficile* is capable of binding the DNA upstream of the open reading frames of *spoIIAA, spoIIE* and *spoIIGA* with low affinity.

We performed EMSA experiments using increasing amounts of purified Spo0A-DBD from *C. difficile* 630Δerm and the DNA fragments indicated above (Fig. 3B). For *spoIIAA* (encoding an anti-anti sigma-factor) and *spoIIE* (encoding a serine phosphatase), we observed a low intensity shifted species at concentrations as low as 150 nM. For *spoIIGA* (encoding a sporulation specific protease) we observed the shifted species only at higher concentrations of protein (>200 nM). The negative control (*spoVG*) did not demonstrate binding of Spo0A-DBD at these concentrations. Moreover, the shift we observed was reversible using unlabeled DNA containing a high affinity binding site, but not using unlabeled DNA that lacked such a site (Figure S1B–D). Therefore, we consider the binding to *spoIIAA*, *spoIIE* and *spoIIGA* genes to be specific, despite the fact that increasing the amount of protein did not seem to cause a significant increase in the amount of DNA in the complex.

Together, these results suggest that Spo0A in *C. difficile* might regulate the transcription of at least a subset of early sporulation genes by direct binding to their promoter regions.

### *C. difficile* Spo0A-DBD Binds to DNA Upstream of *tcdB*

It has previously been reported that the deletion of Spo0A in *C. difficile* results in a significantly lower toxin production and a ∼1000-fold reduction in the toxicity of culture supernatant derived from *spo0A* negative cells towards Vero cells [35]. Considering the absence of a homolog of the *abrB* repressor, direct binding of Spo0A and concomitant activation of toxin gene transcription is a likely mechanism through which this could occur. We found evidence for direct binding of Spo0A-DBD to the region upstream of *tcdB*, encoding one of the major clostridial toxin genes, and possibly *tcdC*, but this did not seem to result in lower toxin levels in our hands.

**Figure 4:**
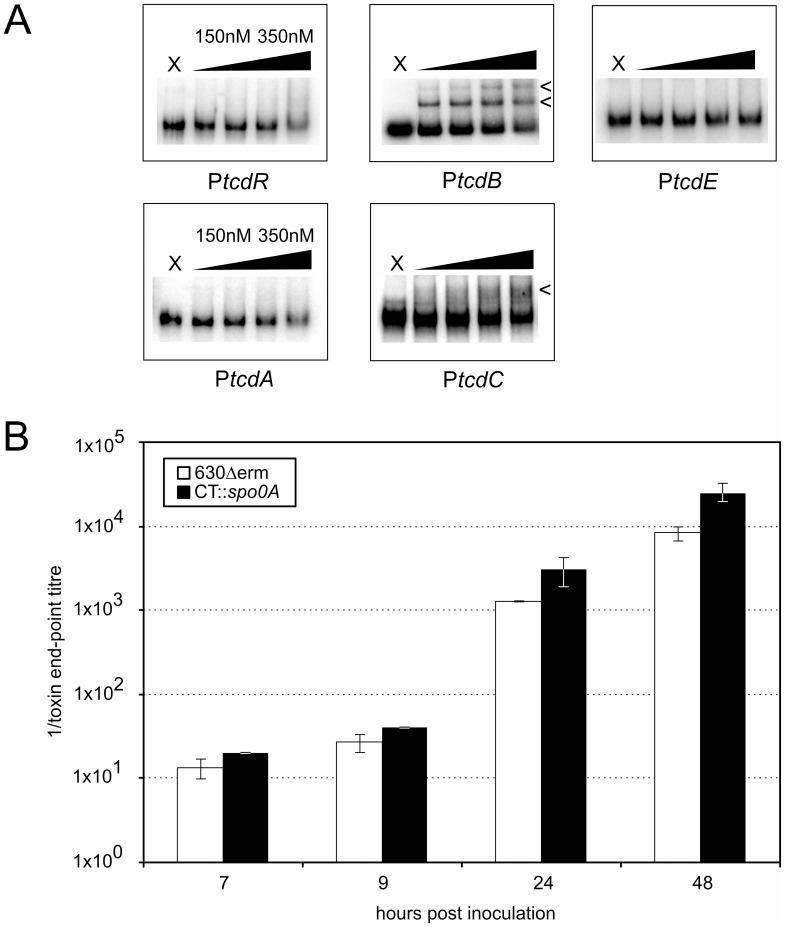
*C. difficile* Spo0A does not contribute to toxin production directly. A. Electrophoretic mobility shift assays using purified *C. difficile* Spo0A-DBD-his6 and radiolabeled DNA fragments corresponding to the upstream regions of the indicated toxin-related genes. X  =  no protein control, the triangle indicates 1.3-fold serial dilutions of protein to the indicated concentrations. Arrows indicate (possible) protein:DNA complexes. **B.** Toxin end-point titre of culture supernatant from wild type (630Δerm; white bars) or *spo0A* mutant (CT::spo0A; black bars) *C. difficile* cells. For details see Materials and Methods. Error bars represent standard error of the mean (n = 3). None of the observed differences were significant in an independent sample t-test.

We performed EMSAs using DNA upstream of *tcdR* (encoding a sigma factor responsible for the activation of toxin gene transcription), *tcdB* (encoding toxin B), *tcdA* (encoding toxin A). In order to test regions upstream of all open reading frames in the PaLoc, we also tested binding of Spo0A to DNA upstream of *tcdE* (encoding a holin-like protein [50], [51]) and *tcdC* (encoding a putative negative regulator of toxin production [52]–[54]), even though this regulator does not have a significant effect on toxin levels under the conditions we used [55], [56]. Of the regions tested, we only observed a clear shifted species, indicative of Spo0A binding, for *tcdB* (Figure 4A); the shifted species in our EMSA assay was reversed by the addition of unlabeled DNA containing a high affinity binding site, but not by DNA lacking such a site (Figure S1E). For *tcdC,* some smearing was observed at all concentrations of proteins tested (Figure 4A), and there did not seem to be a clear effect of the addition of unlabeled DNA fragments (Figure S1F). The probes for *tcdA*, *tcdE* and *tcdR* were indistinguishable from those obtained with our negative control, *spoVG*.

We wanted to determine if toxin levels in culture supernatants were directly or indirectly affected by Spo0A, as was previously suggested. We found no lower toxicity towards Vero cells of culture supernatants derived from *spo0A* mutant cells compared to wild type.

We grew three independent biological replicates of a wild type (630Δerm) or Clostron-generated *spo0A* mutant (CT::*spo0A* - a kind gift of the Minton lab) in glucose-free TTY medium. We harvested culture supernatant at late-exponential phase (approximately 7 hours post inoculation), the transition phase between exponential and stationary growth phase (approximately 9 hours post inoculation), as well as two time points in stationary phase (24 and 48 hours post inoculation) and determined the toxin endpoint titres (see Materials and Methods). In contrast to previous findings, we observed a small (≤4-fold) increase in the toxicity of supernatants derived from *spo0A* mutant cells compared to wild type, but in all cases this difference was not statistically significant (p>0.05, independent sample t-test). In other medium (BHIS), we observed no differences at all (data not shown).

We conclude that Spo0A does not positively affect toxin production in *C. difficile* 630Δerm and the *in vivo* relevance of the binding to regions upstream of *tcdB* and/or *tcdC* is therefore limited under our experimental conditions.

## Discussion

### The Spo0A-box of C. difficile

In *B. subtilis*, the binding site of Spo0A on target DNA has been well-characterized, through a combination of *in vitro* binding assays, determination of *in vivo* binding profiles and mutagenesis of regulated promoter sequences. This work has led to the identification of a conserved core motif, TGTCGAA, or Spo0A box [5], [10], [11], [45]. Depending on the analysis, this motif is flanked by one or more adenine or thymine residues [10], [11]. Interestingly, many target genes do not harbor a perfect match to this consensus sequence, but rather contain one or more degenerate motifs. The differences in these motifs may reflect different promoter architectures (e.g. AT content), modes of action (e.g. activation or repression) or levels of regulation. Spo0A genes in *B. subtilis* can be divided in different classes that respond to different levels of phosphorylated Spo0A [43], [57].

For *C. difficile*, we conclude that the Spo0A protein likely recognizes a motif that is similar to the *B. subtilis* Spo0A box on the basis of four lines of evidence; 1. All DNA binding/contacting residues are conserved (Fig. 2B), 2. *C. difficile* Spo0A can bind with high affinity to a target of *B. subtilis* Spo0A (Fig. 2D), 3. Mutagenesis of key residues in the *B. subtilis* Spo0A box reduces affinity of *C. difficile* Spo0A for DNA (Fig. 2E) and 4. A *B. subtilis* Spo0A box has predictive value for DNA binding by *C. difficile* Spo0A (Fig. 3A). It is conceivable that our model system, using the purified DNA binding domain, does not accurately reflect binding to all target sites, if target site selectivity is determined in part by other parts by of the full length protein. It is likely that differences do exist between the preferred binding sites for both proteins that will be evident when a comprehensive analysis is performed of *in vivo* DNA binding of *C. difficile* Spo0A; based on the limited data set of this study, a MEME analysis [58] already suggests possible differences in the extended Spo0A motif (W.K. Smits, unpublished observations). These differences may relate to the much higher AT content of *C. difficile* compared to *B. subtilis* (71 vs. 56.5%, respectively), or phosphorylation dependent dimerization, for instance.

### Regulation of Sporulation; Feedback Regulation of *spo0A*

The initiation of sporulation in *B. subtilis* is subject to complex regulation (for review see ref [1], [59]). The activation of Spo0A is controlled by a multi-component phosphorelay that can integrate environmental cues [60] and ensures a gradual increase in the level of phosphorylated Spo0A in the cell [57]. In addition, the transcription of the *spo0A* gene is controlled by multiple feedback loops. For instance, Spo0A regulates its own transcription by binding to the *spo0A* promoter [46], as well as by indirectly stimulating the transcription of *sigH*, encoding a sigma factor that recognizes the *spo0A* promoter [48].

In *C. difficile*, there are some interesting differences and similarities in the regulatory pathways. Most notably, there seems to be no phosphorelay [2] and the phosphorylation state of Spo0A is supposedly controlled by orphan histidine kinases [35]. The transcription of *spo0A* in *C. difficile* is under control of the transition state sigma factor Sigma H [37], as it is in *B. subtilis* [61]. Our data indicate that both *spo0A* and *sigH* could be targets for direct regulation by Spo0A in *C. difficile* (Fig. 3A), raising the possibility of auto-regulation of *spo0A*. The putative direct regulation of *sigH* by Spo0A may reflect that the *C. difficile* genome does not harbor a homolog of the pleiotropic regulator AbrB, which is responsible for the Spo0A-dependent regulation of *sigH* in *B. subtilis* [48]. Consistent with a model in which *spo0A* is positively auto-regulated, we noted a sharp increase in the levels of Spo0A as cells approach the stationary growth phase (Figure 1C).

Downstream of Spo0A, we found binding of Spo0A to DNA upstream of several early sporulation genes, such as *spoIIAA*, *spoIIE,* and *spoIIGA* (Fig. 3B). All these observations are consistent with direct regulation of these genes by Spo0A in other organisms [5], [45], [49], [62], and the conservation of the sporulation pathway [2].

### Regulation of Processes Outside Sporulation

Though Spo0A is the key regulator for sporulation in Firmicutes, it regulates numerous other processes in various bacteria. In the non-pathogenic *B. subtilis*, for instance, the protein also affects competence development, biofilm formation, the production of and resistance to antimicrobial compounds, chromosome dynamics and aspects of phage biology [10], [14]–[16]. Importantly, several of these processes are indirectly regulated, through the Spo0A-dependent repression of *abrB*. Additionally, transcription of *abrB* responds already to low levels of Spo0A∼P [43]. As a result these effects are detectable in late-exponential and early stationary phase, as some Spo0A is present throughout growth in *B. subtilis* cells.

Though *abrB* is absent from *C. difficile*, this does not exclude the possibility of indirect transcriptional regulation through Spo0A-dependent effects on other regulators. Alternatively, Spo0A may exert a direct effect. In *Clostridium acetobutylicum* and *C. beijerinckii*, Spo0A is a direct regulator of solvent formation, as well as sporulation [22], [23]. It seems therefore conceivable that Spo0A in *C. difficile* also affects aspects of metabolism. In this respect, it is important to note that also in *C. difficile* Spo0A is detectable from early exponential growth phase on (Figure 1B).

We observed direct binding of *C. difficile* Spo0A to the promoter region of *sigH* (Fig. 3A). This gene encodes the key sigma factor for the transition phase, and regulates processes outside sporulation as well [37]. Moreover, we found significant levels of Spo0A from early stationary phase on (Fig. 1B and unpublished observations), indicating the regulatory actions of Spo0A need not be limited to stationary phase in *C. difficile*. In line with this idea, we found a potential regulatory link between Spo0A and two genes that to our knowledge are not related to the sporulation process, the lipoate ligase *lplA* and the aliphatic sulfonates transporter *ssuA* (Fig. 3A). The presence of a putative Spo0A binding site upstream of these genes, as well as the spacing compared to the start codon, is conserved in the problematic Stoke-Mandeville strain (R20291), a member of PCR ribotype 27. This could indicate that these aspects of regulation by Spo0A are conserved in multiple strains of *C. difficile*.

It should be noted that our work so far has been limited to an *in vitro* analysis of Spo0A binding, and therefore does not indicate whether activation or repression of the putative target genes occurs *in vivo*. To answer this question, detailed transcriptome and/or proteome studies have to be performed. In order to distinguish direct from indirect effects, *in vivo* binding profiles of Spo0A should be performed. The antibodies generated for this study should prove to be useful for this type of experiments.

### Regulation of Toxin Production

Amongst the pathogenic Firmicutes, Spo0A has been reported to affect toxin production in multiple species. In *B. anthracis* a *spo0A* mutation results in elevated levels of AbrB, and concomitantly lower levels of the toxin genes *pagA*, *cya* and *lef* that are under AbrB control [17]. Similarly, the production of the emetic toxin cereulide in *B. cereus* is greatly repressed in a *spo0A* mutant, in an AbrB-dependent manner [63]. In contrast, Spo0A directly represses the expression of the *cry* toxin genes in *B. thuringiensis* and a *spo0A* mutant is therefore a hyper-producer of the insecticidal crystal protein [18], [21]. In *Clostridium perfringens* TpeL, a member of the large clostridial toxins just like TcdA and TcdB, is directly dependent on Spo0A [64] and also the production of enterotoxin in this organism seems to be (indirectly) dependent on sporulation [65], [66].

In *C. difficile* an insertional *spo0A* mutant generated using Clostron technology was reported to have ∼10-fold reduced levels of toxin A (TcdA), both intracellularly and extracellularly as well as ∼1000-fold reduced toxicity towards Vero cells, which are primarily sensitive towards toxin B (TcdB) [35]. Our *in vitro* binding data indicate a potential binding site for Spo0A upstream of *tcdB* and possibly *tcdC* (Fig. 4A). However, the *in vivo* relevance of this binding seems limited as in our hands an independently derived but otherwise identical mutant (a kind gift of the Minton lab; [33]) did not demonstrate a reduced toxicity towards Vero cells. In contrast, we found that in TTY medium toxin levels were slightly elevated in *spo0A* mutant cells compared to wild type (≤2-fold in exponential phase cells up to 4-fold in late-stationary phase cells). The small, and not significant, differences in toxin levels in our experiments might be attributed to differences in the susceptibility of cells for lysis rather than the production of toxin, but could also indicate a negative regulatory effect of Spo0A on toxin production. In support of the latter hypothesis, it was recently reported that a *spo0A* mutant of *C. difficile* strain R20291 (a PCR ribotypes 027/BI/NAP1 epidemic strain) demonstrates ∼10-fold higher toxin levels than its isogenic wild type 30 h post inoculation, and is significantly more virulent in a mouse model of disease [34].

The differences between Underwood *et al* [35] on the one hand and our study as well as the study of Deakin and coworkers [34] on the other hand may be explained by differences in experimental conditions, such as the medium used. However, we observed no difference in cytotoxicity between supernatant derived from wild type or *spo0A* mutant cells when they were grown in BHIS, a medium nearly identical to that used previously (data not shown). Alternatively, the differences could indicate integration of the group II intron at more than one location in the chromosome in the strain used in Underwood *et al* [35]. In the absence of a complementation experiment and/or Southern blot data, this remains to be established.

In summary, our data are consistent with a model in which the regulation of the major clostridial toxins in *C. difficile* is not positively affected by Spo0A, in contrast to previous findings and other pathogenic *Clostridia*. Whether Spo0A is truly a negative regulator of toxin production remains to be confirmed using *in vitro* and *in vivo* transcription assays.

### Concluding Remarks

In the present study we have for the first time demonstrated direct binding of the DNA binding domain of *C. difficile* Spo0A to putative target DNA. This work has revealed that aspects of Spo0A binding are conserved between *Bacillus* and *C. difficile* (0A box, possible auto-regulation and binding to early sporulation promoters), whereas others are not (the absence of *abrB* as a direct target in *C. difficile*, binding to DNA upstream of *lplA*, *ssuA*). The effects of Spo0A on toxin production may be similar to those observed for *B. thuringiensis* [18], [21]. Future work will be aimed at determining the effect of Spo0A on the transcription of the putative target genes, and carry out a comprehensive analysis of Spo0A binding *in vivo*. The identification of genes affected by Spo0A in *C. difficile* may shed light on the role of the protein in virulence and pathogenesis of this organism.

## Supporting Information

Figure S1 **Specificity controls for binding by Spo0A-DBD-his6.** Arrows indicate the position of shifted species (DNA:protein complexes). Titrations with PCR fragments of P*abrB* (containing a high affinity binding site) and P*tcdA* (lacking such a site) correspond to approximately 0.1 nM/µL - 0.03 nM/µL. A. Comparison of binding of Spo0A-DBD-his6, Spo0A-his6 and CD2195-his6 binding to the upstream region of *spoIIAA*. B. Binding of Spo0A-DBD-his6 to the upstream region of *spoIIAA* is reversed by the addition of P*abrB*, but not by the addition of P*tcdA*). C. Binding of Spo0A-DBD-his6 to the upstream region of *spoIIE* is reversed by the addition of P*abrB*, but not by the addition of P*tcdA*. D. Binding of Spo0A-DBD-his6 to the upstream region of *spoIIGA* is reversed by the addition of P*abrB*, but not by the addition of P*tcdA*. E. Binding of Spo0A-DBD-his6 to the upstream region of *tcdB* is reversed by the addition of P*abrB*, but not by the addition of P*tcdA*. F. Binding of Spo0A-DBD-his6 to the upstream region of *tcdC* is not or moderately affected by the addition of P*abrB* and/or P*tcdA*.
(TIF)

Table S1 **Consensus Spo0A boxes in the** ***C. difficile*** **630 genome.**
(PDF)

Text S1 **Oligonucleotides used in this study and PCR cycling conditions for the EMSA probes.**
(PDF)

## References

[1] Errington J (2003) Regulation of endospore formation in *Bacillus subtilis* . Nat Rev Microbiol 1: 117–126. 10.1038/nrmicro750 [doi]. 15035041 10.1038/nrmicro750

[2] Paredes CJ, Alsaker KV, Papoutsakis ET (2005) A comparative genomic view of clostridial sporulation and physiology. Nat Rev Microbiol 3: 969–978. nrmicro1288 [pii];10.1038/nrmicro1288 [doi]. 16261177 10.1038/nrmicro1288

[3] Ferrari FA, Trach K, LeCoq D, Spence J, Ferrari E, et al (1985) Characterization of the spo0A locus and its deduced product. Proc Natl Acad Sci U S A 82: 2647–2651. 3157992 10.1073/pnas.82.9.2647 PMC397621

[4] Bird TH, Grimsley JK, Hoch JA, Spiegelman GB (1993) Phosphorylation of Spo0A activates its stimulation of *in vitro* transcription from the *Bacillus subtilis spoIIG* operon. Mol Microbiol 9: 741–749. 8231806 10.1111/j.1365-2958.1993.tb01734.x

[5] Baldus JM, Green BD, Youngman P, Moran CP Jr (1994) Phosphorylation of *Bacillus subtilis* transcription factor Spo0A stimulates transcription from the *spoIIG* promoter by enhancing binding to weak 0A boxes. J Bacteriol 176: 296–306. 8288522 10.1128/jb.176.2.296-306.1994 PMC205050

[6] Burbulys D, Trach KA, Hoch JA (1991) Initiation of sporulation in *B. subtilis* is controlled by a multicomponent phosphorelay. Cell 64: 545–552. 0092–8674(91)90238-T [pii]. 1846779 10.1016/0092-8674(91)90238-t

[7] Grimsley JK, Tjalkens RB, Strauch MA, Bird TH, Spiegelman GB, et al (1994) Subunit composition and domain structure of the Spo0A sporulation transcription factor of *Bacillus subtilis* . J Biol Chem 269: 16977–16982. 8207022

[8] Lewis RJ, Scott DJ, Brannigan JA, Ladds JC, Cervin MA, et al (2002) Dimer formation and transcription activation in the sporulation response regulator Spo0A. J Mol Biol 316: 235–245. 10.1006/jmbi.2001.5331 [doi];S0022283601953318 [pii]. 11851334 10.1006/jmbi.2001.5331

[9] Muchova K, Lewis RJ, Perecko D, Brannigan JA, Ladds JC, et al (2004) Dimer-induced signal propagation in Spo0A. Mol Microbiol 53: 829–842. 10.1111/j.1365–2958.2004.04171.x [doi];MMI4171 [pii]. 15255896 10.1111/j.1365-2958.2004.04171.x

[10] Molle V, Fujita M, Jensen ST, Eichenberger P, Gonzalez-Pastor JE, et al (2003) The Spo0A regulon of *Bacillus subtilis* . Mol Microbiol 50: 1683–1701. 3818 [pii]. 14651647 10.1046/j.1365-2958.2003.03818.x

[11] Liu J, Tan K, Stormo GD (2003) Computational identification of the Spo0A-phosphate regulon that is essential for the cellular differentiation and development in Gram-positive spore-forming bacteria. Nucleic Acids Res 31: 6891–6903. 14627822 10.1093/nar/gkg879 PMC290249

[12] Lewis RJ, Krzywda S, Brannigan JA, Turkenburg JP, Muchova K, et al (2000) The trans-activation domain of the sporulation response regulator Spo0A revealed by X-ray crystallography. Mol Microbiol 38: 198–212. mmi2134 [pii]. 11069648 10.1046/j.1365-2958.2000.02134.x

[13] Zhao H, Msadek T, Zapf J, Madhusudan, Hoch JA, et al (2002) DNA complexed structure of the key transcription factor initiating development in sporulating bacteria. Structure 10: 1041–1050. S0969212602008031 [pii]. 12176382 10.1016/s0969-2126(02)00803-1

[14] Castilla-Llorente V, Munoz-Espin D, Villar L, Salas M, Meijer WJ (2006) Spo0A, the key transcriptional regulator for entrance into sporulation, is an inhibitor of DNA replication. EMBO J 25: 3890–3899. 7601266 [pii];10.1038/sj.emboj.7601266 [doi]. 16888621 10.1038/sj.emboj.7601266 PMC1553192

[15] Hahn J, Roggiani M, Dubnau D (1995) The major role of Spo0A in genetic competence is to downregulate *abrB*, an essential competence gene. J Bacteriol 177: 3601–3605. 7768874 10.1128/jb.177.12.3601-3605.1995 PMC177070

[16] Hamon MA, Lazazzera BA (2001) The sporulation transcription factor Spo0A is required for biofilm development in *Bacillus subtilis* . Mol Microbiol 42: 1199–1209. 2709 [pii]. 11886552 10.1046/j.1365-2958.2001.02709.x

[17] Saile E, Koehler TM (2002) Control of anthrax toxin gene expression by the transition state regulator *abrB* . J Bacteriol 184: 370–380. 11751813 10.1128/JB.184.2.370-380.2002 PMC139583

[18] Agaisse H, Lereclus D (1994) Expression in *Bacillus subtilis* of the *Bacillus thuringiensis cryIIIA* toxin gene is not dependent on a sporulation-specific sigma factor and is increased in a *spo0A* mutant. J Bacteriol 176: 4734–4741. 8045904 10.1128/jb.176.15.4734-4741.1994 PMC196296

[19] Baum JA, Malvar T (1995) Regulation of insecticidal crystal protein production in *Bacillus thuringiensis* . Mol Microbiol 18: 1–12. 8596449 10.1111/j.1365-2958.1995.mmi_18010001.x

[20] Chen HJ, Tsai TK, Pan SC, Lin JS, Tseng CL, et al (2010) The master transcription factor Spo0A is required for poly(3-hydroxybutyrate) (PHB) accumulation and expression of genes involved in PHB biosynthesis in *Bacillus thuringiensis* . FEMS Microbiol Lett 304: 74–81. FML1888 [pii];10.1111/j.1574–6968.2010.01888.x [doi]. 20100285 10.1111/j.1574-6968.2010.01888.x

[21] Poncet S, Dervyn E, Klier A, Rapoport G (1997) Spo0A represses transcription of the *cry* toxin genes in *Bacillus thuringiensis* . Microbiology 143 (Pt 8): 2743–2751. 10.1099/00221287-143-8-2743 9274027

[22] Ravagnani A, Jennert KC, Steiner E, Grunberg R, Jefferies JR, et al (2000) Spo0A directly controls the switch from acid to solvent production in solvent-forming clostridia. Mol Microbiol 37: 1172–1185. mmi2071 [pii]. 10972834 10.1046/j.1365-2958.2000.02071.x

[23] Harris LM, Welker NE, Papoutsakis ET (2002) Northern, morphological, and fermentation analysis of *spo0A* inactivation and overexpression in *Clostridium acetobutylicum* ATCC 824. J Bacteriol 184: 3586–3597. 12057953 10.1128/JB.184.13.3586-3597.2002 PMC135115

[24] Rupnik M, Wilcox MH, Gerding DN (2009) *Clostridium difficile* infection: new developments in epidemiology and pathogenesis. Nat Rev Microbiol 7: 526–536. nrmicro2164 [pii];10.1038/nrmicro2164 [doi]. 19528959 10.1038/nrmicro2164

[25] Vedantam G, Clark A, Chu M, McQuade R, Mallozzi M, et al (2012) *Clostridium difficile* infection: Toxins and non-toxin virulence factors, and their contributions to disease establishment and host response. Gut Microbes 3. 19399 [pii]. 10.4161/gmic.19399 PMC3370945 22555464

[26] Freeman J, Bauer MP, Baines SD, Corver J, Fawley WN, et al (2010) The changing epidemiology of *Clostridium difficile* infections. Clin Microbiol Rev 23: 529–549. 23/3/529 [pii];10.1128/CMR.00082-09 [doi]. 20610822 10.1128/CMR.00082-09 PMC2901659

[27] Goorhuis A, Bakker D, Corver J, Debast SB, Harmanus C, et al (2008) Emergence of *Clostridium difficile* infection due to a new hypervirulent strain, polymerase chain reaction ribotype 078. Clin Infect Dis 47: 1162–1170. 10.1086/592257 [doi]. 18808358 10.1086/592257

[28] Warny M, Pepin J, Fang A, Killgore G, Thompson A, et al (2005) Toxin production by an emerging strain of *Clostridium difficile* associated with outbreaks of severe disease in North America and Europe. Lancet 366: 1079–1084. S0140–6736(05)67420-X [pii];10.1016/S0140–6736(05)67420-X [doi]. 16182895 10.1016/S0140-6736(05)67420-X

[29] Lyras D, O'Connor JR, Howarth PM, Sambol SP, Carter GP, et al (2009) Toxin B is essential for virulence of *Clostridium difficile* . Nature 458: 1176–1179. nature07822 [pii];10.1038/nature07822 [doi]. 19252482 10.1038/nature07822 PMC2679968

[30] Kuehne SA, Cartman ST, Heap JT, Kelly ML, Cockayne A, et al (2010) The role of toxin A and toxin B in *Clostridium difficile* infection. Nature 467: 711–713. nature09397 [pii];10.1038/nature09397 [doi]. 20844489 10.1038/nature09397

[31] Perelle S, Gibert M, Bourlioux P, Corthier G, Popoff MR (1997) Production of a complete binary toxin (actin-specific ADP-ribosyltransferase) by *Clostridium difficile* CD196. Infect Immun 65: 1402–1407. 9119480 10.1128/iai.65.4.1402-1407.1997 PMC175146

[32] Stubbs S, Rupnik M, Gibert M, Brazier J, Duerden B, et al (2000) Production of actin-specific ADP-ribosyltransferase (binary toxin) by strains of *Clostridium difficile* . FEMS Microbiol Lett 186: 307–312. S0378-1097(00)00162-2 [pii]. 10802189 10.1111/j.1574-6968.2000.tb09122.x

[33] Heap JT, Pennington OJ, Cartman ST, Carter GP, Minton NP (2007) The ClosTron: a universal gene knock-out system for the genus *Clostridium* . J Microbiol Methods 70: 452–464. S0167-7012(07)00208-4 [pii];10.1016/j.mimet.2007.05.021 [doi]. 17658189 10.1016/j.mimet.2007.05.021

[34] Deakin LJ, Clare S, Fagan RP, Dawson LF, Pickard DJ, et al (2012) *Clostridium difficile spo0A* gene is a persistence and transmission factor. Infect Immun. IAI.00147-12 [pii];10.1128/IAI.00147-12 [doi]. 10.1128/IAI.00147-12 PMC3434595 22615253

[35] Underwood S, Guan S, Vijayasubhash V, Baines SD, Graham L, et al (2009) Characterization of the sporulation initiation pathway of *Clostridium difficile* and its role in toxin production. J Bacteriol 191: 7296–7305. JB.00882-09 [pii];10.1128/JB.00882-09 [doi]. 19783633 10.1128/JB.00882-09 PMC2786572

[36] Steiner E, Dago AE, Young DI, Heap JT, Minton NP, et al (2011) Multiple orphan histidine kinases interact directly with Spo0A to control the initiation of endospore formation in *Clostridium acetobutylicum* . Mol Microbiol 80: 641–654. 10.1111/j.1365-2958.2011.07608.x [doi]. 21401736 10.1111/j.1365-2958.2011.07608.x PMC3097173

[37] Saujet L, Monot M, Dupuy B, Soutourina O, Martin-Verstraete I (2011) The key sigma factor of transition phase, SigH, controls sporulation, metabolism, and virulence factor expression in *Clostridium difficile* . J Bacteriol 193: 3186–3196. JB.00272-11 [pii];10.1128/JB.00272-11 [doi]. 21572003 10.1128/JB.00272-11 PMC3133256

[38] Hussain HA, Roberts AP, Mullany P (2005) Generation of an erythromycin-sensitive derivative of *Clostridium difficile* strain 630 (630Deltaerm) and demonstration that the conjugative transposon Tn916DeltaE enters the genome of this strain at multiple sites. J Med Microbiol 54: 137–141. 15673506 10.1099/jmm.0.45790-0

[39] Snijder EJ, Bredenbeek PJ, Dobbe JC, Thiel V, Ziebuhr J, et al (2003) Unique and conserved features of genome and proteome of SARS-coronavirus, an early split-off from the coronavirus group 2 lineage. J Mol Biol 331: 991–1004. S0022283603008659 [pii]. 12927536 10.1016/S0022-2836(03)00865-9 PMC7159028

[40] Baerends RJ, Smits WK, de Jong A, Hamoen LW, Kok J, et al (2004) Genome2D: a visualization tool for the rapid analysis of bacterial transcriptome data. Genome Biol 5: R37. 10.1186/gb-2004-5-5-r37 [doi];gb-2004-5-5-r37 [pii]. 15128451 10.1186/gb-2004-5-5-r37 PMC416473

[41] Heap JT, Cartman ST, Kuehne SA, Cooksley C, Minton NP, (2010) ClosTron-targeted mutagenesis. Methods Mol Biol 646: 165-182. 10.1007/978-1-60327-365-7_11 [doi]. 20597009 10.1007/978-1-60327-365-7_11

[42] Sebaihia M, Wren BW, Mullany P, Fairweather NF, Minton N, et al (2006) The multidrug-resistant human pathogen *Clostridium difficile* has a highly mobile, mosaic genome. Nat Genet 38: 779–786. ng1830 [pii];10.1038/ng1830 [doi]. 16804543 10.1038/ng1830

[43] Fujita M, Gonzalez-Pastor JE, Losick R (2005) High- and low-threshold genes in the Spo0A regulon of *Bacillus subtilis* . J Bacteriol 187: 1357–1368. 187/4/1357 [pii];10.1128/JB.187.4.1357-1368.2005 [doi]. 15687200 10.1128/JB.187.4.1357-1368.2005 PMC545642

[44] Strauch M, Webb V, Spiegelman G, Hoch JA (1990) The SpoOA protein of *Bacillus subtilis* is a repressor of the *abrB* gene. Proc Natl Acad Sci U S A 87: 1801–1805. 2106683 10.1073/pnas.87.5.1801 PMC53571

[45] Baldus JM, Buckner CM, Moran CP Jr (1995) Evidence that the transcriptional activator Spo0A interacts with two sigma factors in *Bacillus subtilis* . Mol Microbiol 17: 281–290. 7494477 10.1111/j.1365-2958.1995.mmi_17020281.x

[46] Strauch MA, Trach KA, Day J, Hoch JA (1992) Spo0A activates and represses its own synthesis by binding at its dual promoters. Biochimie 74: 619–626. 1391039 10.1016/0300-9084(92)90133-y

[47] Weir J, Predich M, Dubnau E, Nair G, Smith I (1991) Regulation of *spo0H*, a gene coding for the *Bacillus subtilis* sigma H factor. J Bacteriol 173: 521–529. 1898930 10.1128/jb.173.2.521-529.1991 PMC207041

[48] Fujita M, Sadaie Y (1998) Feedback loops involving Spo0A and AbrB in *in vitro* transcription of the genes involved in the initiation of sporulation in *Bacillus subtilis* . J Biochem 124: 98–104. 9644251 10.1093/oxfordjournals.jbchem.a022103

[49] York K, Kenney TJ, Satola S, Moran CP Jr, Poth H, et al (1992) Spo0A controls the sigma A-dependent activation of *Bacillus subtilis* sporulation-specific transcription unit *spoIIE* . J Bacteriol 174: 2648–2658. 1556084 10.1128/jb.174.8.2648-2658.1992 PMC205905

[50] Olling A, Seehase S, Minton NP, Tatge H, Schroter S, et al (2012) Release of TcdA and TcdB from *Clostridium difficile* cdi 630 is not affected by functional inactivation of the *tcdE* gene. Microb Pathog 52: 92–100. S0882-4010(11)00191-4 [pii];10.1016/j.micpath.2011.10.009 [doi]. 22107906 10.1016/j.micpath.2011.10.009

[51] Tan KS, Wee BY, Song KP (2001) Evidence for holin function of *tcdE* gene in the pathogenicity of *Clostridium difficile* . J Med Microbiol 50: 613–619. 11444771 10.1099/0022-1317-50-7-613

[52] Matamouros S, England P, Dupuy B (2007) *Clostridium difficile* toxin expression is inhibited by the novel regulator TcdC. Mol Microbiol 64: 1274–1288. MMI5739 [pii];10.1111/j.1365-2958.2007.05739.x [doi]. 17542920 10.1111/j.1365-2958.2007.05739.x

[53] Dupuy B, Govind R, Antunes A, Matamouros S (2008) *Clostridium difficile* toxin synthesis is negatively regulated by TcdC. J Med Microbiol 57: 685–689. 57/6/685 [pii];10.1099/jmm.0.47775-0 [doi]. 18480323 10.1099/jmm.0.47775-0

[54] Carter GP, Douce GR, Govind R, Howarth PM, Mackin KE, et al (2011) The anti-sigma factor TcdC modulates hypervirulence in an epidemic BI/NAP1/027 clinical isolate of *Clostridium difficile* . PLoS Pathog 7: e1002317. 10.1371/journal.ppat.1002317 [doi];PPATHOGENS-D-11-00972 [pii]. 22022270 10.1371/journal.ppat.1002317 PMC3192846

[55] Cartman ST, Kelly ML, Heeg D, Heap JT, Minton NP (2012) Precise Manipulation of the *Clostridium difficile* Chromosome Reveals a Lack of Association Between *tcdC* Genotype and Toxin Production. Appl Environ Microbiol. AEM.00249-12 [pii];10.1128/AEM.00249-12 [doi]. 10.1128/AEM.00249-12 PMC3370502 22522680

[56] Bakker D, Smits WK, Kuijper EJ, Corver J (2012) TcdC Does Not Significantly Repress Toxin Expression in *Clostridium difficile* 630ΔErm. PLoS ONE 7(8): e43247 doi:10.1371/journal.pone.0043247 22912837 10.1371/journal.pone.0043247 PMC3422341

[57] Fujita M, Losick R (2005) Evidence that entry into sporulation in *Bacillus subtilis* is governed by a gradual increase in the level and activity of the master regulator Spo0A. Genes Dev 19: 2236–2244. 19/18/2236 [pii];10.1101/gad.1335705 [doi]. 16166384 10.1101/gad.1335705 PMC1221893

[58] Bailey TL, Boden M, Buske FA, Frith M, Grant CE, et al (2009) MEME SUITE: tools for motif discovery and searching. Nucleic Acids Res 37: W202–W208. gkp335 [pii];10.1093/nar/gkp335 [doi]. 19458158 10.1093/nar/gkp335 PMC2703892

[59] Piggot PJ, Hilbert DW (2004) Sporulation of *Bacillus subtilis* . Curr Opin Microbiol 7: 579–586. S1369-5274(04)00125-0 [pii];10.1016/j.mib.2004.10.001 [doi]. 15556029 10.1016/j.mib.2004.10.001

[60] Ireton K, Rudner DZ, Siranosian KJ, Grossman AD (1993) Integration of multiple developmental signals in *Bacillus subtilis* through the Spo0A transcription factor. Genes Dev 7: 283–294. 8436298 10.1101/gad.7.2.283

[61] Predich M, Nair G, Smith I (1992) *Bacillus subtilis* early sporulation genes *kinA*, *spo0F*, and *spo0A* are transcribed by the RNA polymerase containing sigma H. J Bacteriol. 174: 2771–2778. 10.1128/jb.174.9.2771-2778.1992 PMC205927 1569009

[62] Satola S, Kirchman PA, Moran CP Jr (1991) Spo0A binds to a promoter used by sigma A RNA polymerase during sporulation in *Bacillus subtilis* . Proc Natl Acad Sci U S A 88: 4533–4537. 1903544 10.1073/pnas.88.10.4533 PMC51695

[63] Lucking G, Dommel MK, Scherer S, Fouet A, Ehling-Schulz M (2009) Cereulide synthesis in emetic *Bacillus cereus* is controlled by the transition state regulator AbrB, but not by the virulence regulator PlcR. Microbiology 155: 922–931. 155/3/922 [pii];10.1099/mic.0.024125-0 [doi]. 19246763 10.1099/mic.0.024125-0

[64] Paredes-Sabja D, Sarker N, Sarker MR (2011) *Clostridium perfringens tpeL* is expressed during sporulation. Microb Pathog 51: 384–388. S0882-4010(11)00123-9 [pii];10.1016/j.micpath.2011.05.006 [doi]. 21810463 10.1016/j.micpath.2011.05.006

[65] Duncan CL, Strong DH, Sebald M (1972) Sporulation and enterotoxin production by mutants of *Clostridium perfringens* . J Bacteriol 110: 378–391. 4336110 10.1128/jb.110.1.378-391.1972 PMC247421

[66] Zhao Y, Melville SB (1998) Identification and characterization of sporulation-dependent promoters upstream of the enterotoxin gene (*cpe*) of *Clostridium perfringens* . J Bacteriol 180: 136–142. 9422603 10.1128/jb.180.1.136-142.1998 PMC106859

