# Supplementary figures and images for: C. difficile 630Δerm Spo0A Regulates Sporulation, but Does Not Contribute to Toxin Production, by Direct High-Affinity Binding to Target DNA
Source: PLoS One. 2012 Oct 31;7(10):e48608. doi: 10.1371/journal.pone.0048608 (PMC3485338; doi:10.1371/journal.pone.0048608)

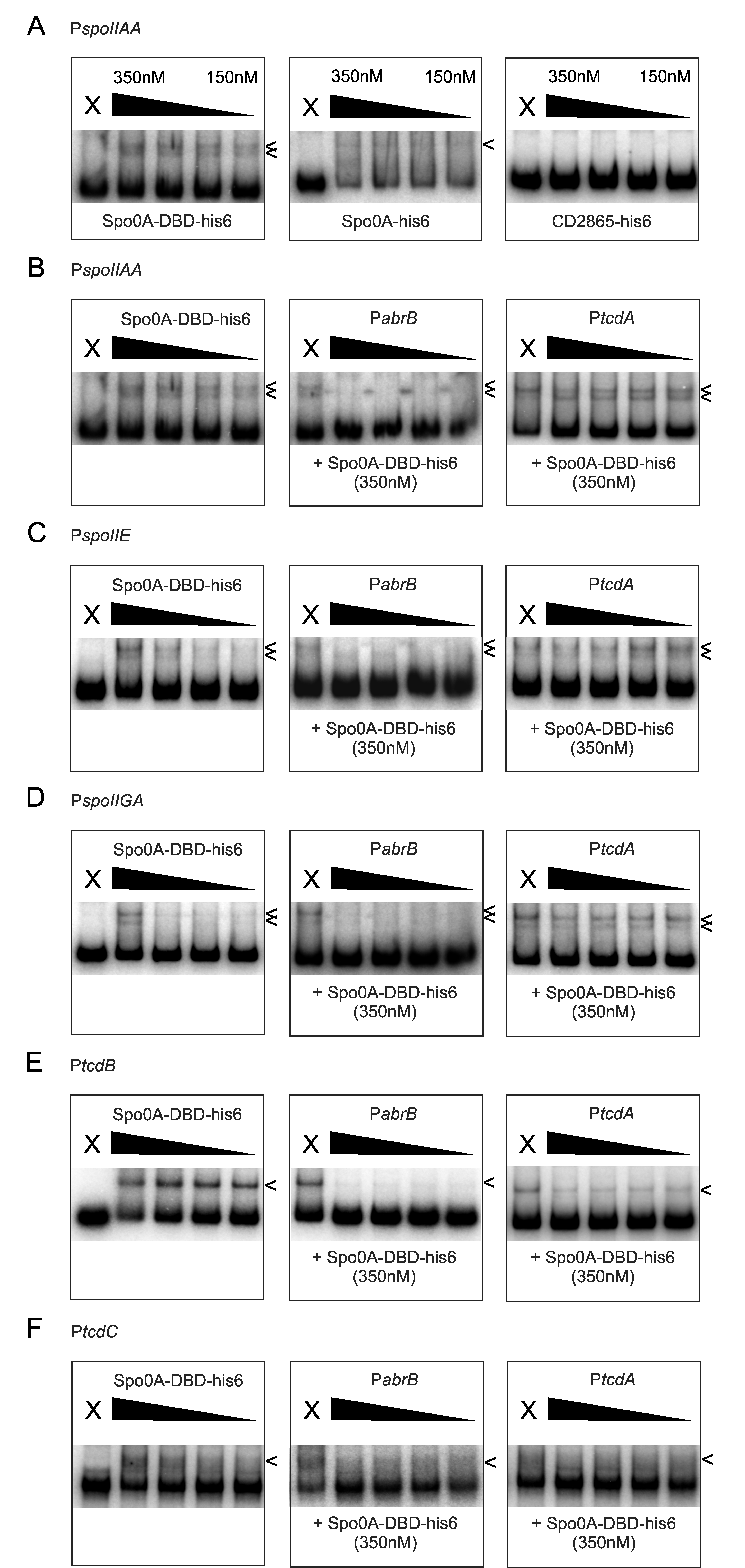

Supplement: Figure S1 — Specificity controls for binding by Spo0A-DBD-his6. Arrows indicate the position of shifted species (DNA:protein complexes). Titrations with PCR fragments of PabrB (containing a high affinity binding site) and PtcdA (lacking such a site) correspond to approximately 0.1 nM/µL - 0.03 nM/µL. A. Comparison of binding of Spo0A-DBD-his6, Spo0A-his6 and CD2195-his6 binding to the upstream region of spoIIAA. B. Binding of Spo0A-DBD-his6 to the upstream region of spoIIAA is reversed by the addition of PabrB, but not by the addition of PtcdA). C. Binding of Spo0A-DBD-his6 to the upstream region of spoIIE is reversed by the addition of PabrB, but not by the addition of PtcdA. D. Binding of Spo0A-DBD-his6 to the upstream region of spoIIGA is reversed by the addition of PabrB, but not by the addition of PtcdA. E. Binding of Spo0A-DBD-his6 to the upstream region of tcdB is reversed by the addition of PabrB, but not by the addition of PtcdA. F. Binding of Spo0A-DBD-his6 to the upstream region of tcdC is not or moderately affected by the addition of PabrB and/or PtcdA. (TIF) [file pone.0048608.s001.tif]
