# Supplementary material for: C. difficile 630Δerm Spo0A Regulates Sporulation, but Does Not Contribute to Toxin Production, by Direct High-Affinity Binding to Target DNA
Source: PLoS One. 2012 Oct 31;7(10):e48608. doi: 10.1371/journal.pone.0048608 (PMC3485338; doi:10.1371/journal.pone.0048608)
Supplement: Table S1 — Consensus Spo0A boxes in the C. difficile 630 genome. (PDF) [file pone.0048608.s002.pdf]

Rosenbusch *et al* (*C. difficile* 630Δerm Spo0A regulates sporulation, but does not contribute to toxin production, by direct high-affinity binding to target DNA)
Supplemental Table 1

Box position	Strand	Start position downstream gene	Downstream Gene	Distance to downstream gene	Start position upstream gene	UpStream Gene	Distance to upstream gene
82910	+	82988	sigH	78	82408	CD0055	502
157222	-	158247	CD0114	1025	157156	ptb	66
209883	+	211074	CD0160	1191	208307	CD0159	1576
217452	+	223261	CD0168	5809	216895	CD0163B	557
269100	+	270469	CD0206	1369	268186	CD0204	914
304483	-	304766	CD0241	283	304049	CD0240	434
357034	+	357416	CD0296	382	356025	CD0294	1009
447054	+	457410	bglF	10356	444740	CD0373	2314
457994	+	459275	bglA	1281	457163	CD0354A	831
485587	-	486450	CD0416	863	485550	CD0414	37
507073	+	507601	CD0427	528	506947	CD0425	126
520281	+	520353	CD0438	72	520127	CD0437	154
571711	-	579018	sugE	7307	571611	spaK	100
575610	-	579018	sugE	3408	574990	CD0484	620
592437	+	593007	orf15	570	590576	orf17	1861
598093	+	598817	orf13	724	595175	orf15	2918
636986	+	637587	CD0532	601	635577	CD0530	1409
669408	+	670855	CD0563	1447	669167	CD0561	241
704836	+	705087	CD0588	251	704154	CD0586	682
708506	+	718446	CD0594	9940	708270	CD0589	236
720090	-	720829	CD0596	739	719759	CD0594	331
726069	-	726099	CD0604	30	725673	CD0603	396
742805	-	744929	CD0623	2124	742122	CD0617	683
756338	+	757585	CD0634	1247	755964	CD0632	374
840429	-	842615	CD0694	2186	839555	CD0691	874
870080	+	870253	CD0712	173	869999	argS	81
870648	+	871523	CD0713	875	869999	argS	649
871097	+	871523	CD0713	426	869999	argS	1098
902249	+	903936	glpK1	1687	902130	CD0739	119
907049	-	31212	tRNA-Ser (	-875837	906618	CD0742	431
1091235	-	1094326	CD0907A	3091	1144398	CD0904	-53163
1112262	+	1112324	CD0942	62	1112200	CD0941	62
1151357	+	1152017	CD0988	660	1151159	CD0986	198
1156662	+	1157903	CD0993	1241	1156463	leuD	199
1159813	+	1160107	serA	294	1158418	CD0993	1395
1193281	-	1194085	CD1022	804	1191708	CD1020	1573
1226345	-	1227866	addA	1521	1224409	CD1039	1936
1300455	-	1315171	CD1119	14716	1300306	CD1105	149
1320011	+	1320105	CD1123	94	1319941	CD1122	70
1341693	-	1341765	maf	72	1341651	CD1142A	42
1356743	+	1357131	CD1158	388	1355407	CD1156	1336
1369508	+	1370023	etfB3	515	1368623	CD1169	885
1374822	-	1375461	ackA	639	1373456	CD1173	1366
1412522	+	1412597	spo0A	75	1412455	spoIVB	67
1423277	-	1424420	CD1226	1143	1422678	deoD	599
1519722	+	1520506	rbfA	784	1518501	CD1308	1221
1556522	+	1557253	exoA	731	1555758	CD1339	764
1564946	+	1565100	CD1348	154	1563935	CD1346	1011
1571633	-	1571945	cspB	312	1570357	CD1353	1276
1631740	+	1633314	CD1409	1574	1630626	CD1406	1114
1719544	+	1719626	ssuA	82	1718901	ssuC	643
1765378	+	1765767	CD1523	389	1765351	CD1522	27
1795018	-	1795799	hisZ	781	1794937	CD1546	81
1804607	+	1804700	CD1556	93	1803947	CD1555	660
1818105	+	1818353	CD1570	248	1818065	CD1569	40
1862947	+	1863245	CD1610	298	1862028	CD1608	919
1871333	+	1871735	CD1616	402	1871053	CD1615	280
1918749	-	1919166	CD1655	417	1918682	lplA	67
1975325	+	1976366	thiC	1041	1973099	ribD	2226
1997328	-	1999657	CD1726	2329	1997118	CD1722	210
2045661	+	2045829	CD1768	168	2045434	gapA	227
2082171	-	2083078	CD1802	907	2081633	CD1800	538
2143812	-	2144675	CD1854	863	2143774	CD1852	38
2206042	+	2208126	CD1906	2084	2206011	CD1904	31
2280366	-	2286967	CD1984	6601	2279284	mutL	1082
2292543	-	2296885	cspC	4342	2291200	CD1986	1343
2422679	-	2433380	CD2106	10701	2421644	CD2095	1035
2573691	-	2574631	CD2223	940	2572879	CD2221	812
2574754	-	2575127	CD2224	373	2573933	CD2222	821
2598894	-	2607067	CD2250	8173	2598370	asnS	524
2654769	-	2658954	CD2291	4185	2654595	CD2287	174
2660564	-	2660635	CD2295	71	2660499	CD2294	65
2662716	-	2669195	CD2305	6479	2662453	CD2297	263
2742084	-	2756823	CD2387	14739	2741789	CD2375	295
2825168	-	2833085	CD2456	7917	2824441	CD2449	727
2879888	+	2882690	CD2498	2802	2879224	selB	664
2901243	-	2903694	aspD	2451	2901059	CD2512	184
2968575	+	2968663	CD2566	88	2968429	CD2565	146
3005718	-	3012145	CD2603	6427	3005392	CD2596	326
3031261	-	3032001	CD2625	740	3030984	CD2623	277
3037443	+	3072487	CD2662	35044	3037399	CD2628	44
3040911	+	3072487	CD2662	31576	3040406	glyC	505
3198281	-	3211405	CD2763	13124	3196569	CD2752	1712
3235190	+	3244556	CD2788	9366	3234142	mviN	1048
3373219	-	3375865	CD2887	2646	3373010	licB	209
3410385	-	3410441	CD2925	56	3410317	CD2924	68
3439651	-	3448377	CD2966A	8726	3439008	CD2961	643
3528667	+	3534919	CD3042	6252	3527215	CD3036	1452

3670291	-	3672294	CD3148	2003	3669474	CD3146A	817
3726956	+	3731127	CD3188	4171	3726406	dpaL2	550
3785971	-	3804291	sspB	18320	3784355	CD3232	1616
3954157	-	250260	cls	-3703897	3952771	CD3380	1386
3981796	+	250260	cls	-3731536	3980827	murB	969
3997802	+	4031482	CD3440	33680	3996171	uvrA	1631
3998065	+	4031482	CD3440	33417	3996171	uvrA	1894
4043207	-	4044971	CD3452	1764	4042271	agaS	936
4154381	+	4179603	CD3579	25222	4154219	lysS	162
4154862	-	4179603	CD3579	24741	4154713	greA	149
4161384	-	4179603	CD3579	18219	4159428	birA	1956
4212360	-	4217582	CD3613	5222	4212218	CD3604	142
4263250	-	4264158	CD3654	908	4262909	CD3652	341
4276257	+	4278618	CD3669	2361	4276008	CD3665	249
