# Supplementary material for: C. difficile 630Δerm Spo0A Regulates Sporulation, but Does Not Contribute to Toxin Production, by Direct High-Affinity Binding to Target DNA
Source: PLoS One. 2012 Oct 31;7(10):e48608. doi: 10.1371/journal.pone.0048608 (PMC3485338; doi:10.1371/journal.pone.0048608)
Supplement: Text S1 — Oligonucleotides used in this study and PCR cycling conditions for the EMSA probes. (PDF) [file pone.0048608.s003.pdf]

Rosenbusch *et al*
Supplemental Text S1

Oligonucleotides used in this study and PCR cycling conditions for the EMSA probes. Starred PCR products have cycling conditions listed below.

Primer	Sequence	Fragment
oWKS-24	5′- GTGAGCGGATAACAATTTCACACAGG – 3′	pCR2.1TOPO insert
oWKS-25	5′- GGTTTTCCCAGTCACGACGTTGTAA – 3′	pCR2.1TOPO insert
oWKS-135	5′- GCGAAATTAATACGACTCACTATAGG – 3′	pET21b(+) insert
oWKS-136	5′- CAGCCAACTCAGCTTCCTTTC – 3′	pET21b(+) insert
oWKS-1223a	5′- TGCTCGAGTTTAACCATACTATGTTCTAGT - 3′	*C. difficile spo0A*-DBD
oWKS-1124	5′- CTCATATGACTAGAAGTGATTTTGTTAAG - 3′	*C. difficile spo0A* and *spo0A*-DBD
oWKS-1122	5′- TTTCATATGGGGGGATTTTTAGTGG – 3′	*C. difficile spo0A*
oWKS-1193	5′ – AGGATTTTTAGTAGGATAATAGC – 3′	**B. subtilis* P*abrB*
oWKS-1194	5′ - CTCCCAAGAGATACTTATTTG - 3′	**B. subtilis* P*abrB*
oWKS-1223	5′ - CTCCCAAGAGATACTTATTTGTTTAAATTATATTTTTCTTCGTCATTATTCAACAAAATC - 3′	**B. subtilis* P*abrB* -C4A
oWKS-1224	5′ - CTCCCAAGAGATACTTATTTGTTTAAATTATATTTTTCTTCGTCATTATTAAACAAAATC - 3′	**B. subtilis* P*abrB* -C4A-G5A
oWKS-1225	5′ - CTCCCAAGAGATACTTATTTGTTTAAATTATATTTTTCTTCGTCATTATTCGAAAAAATC - 3′	**B. subtilis* P*abrB* -G2A

oWKS-1226	5′ - CTCCCAAGAGATACTTATTTGTTTAAATTATATTTTTCTTCGTCATTATTCAAAAAAATC - 3′	**B. subtilis* P*abrB* -G2A-C4A
oWKS-1230	5′ - CTCCCAAGAGATACTTATTTGTTTAAATTATATTTTTCTTCGTCATTATTAGACAAAATC - 3′	**B. subtilis* P*abrB* -G5A
oWKS-1199	5′ - TGAAAGCGGAGGATACGAAG - 3′	**B. subtilis* P*citG*
oWKS-1200	5′ - CAATTCTGTATTCCATTTATGTATC - 3′	**B. subtilis* P*citG*
oWKS-1166	5′ - ACACAGGAGGTATCGTACAG - 3′	**C. difficile* P*spo0A*
oWKS-1167	5′ - CCCCCCATTAAAAAACATCTTC - 3′	**C. difficile* P*spo0A*
oWKS-1227	5′ - GGTGCTACTAGAATGTCTGC - 3′	**C. difficile* P*sigH*
oWKS-1228	5′ - GACAATTGTCCACTAACTCATAAC - 3′	**C. difficile* P*sigH*
oWKS-1231	5′ - GCCTCTACCCTTCTATTCAC - 3′	**C. difficile* P*lplA*
oWKS-1232	5′ - TAACATTAAAGTAGTTCCTCCATAG - 3′	**C. difficile* P*lplA*
oWKS-1235	5′ - GCTGATGGACGAACCATTTG - 3′	**C. difficile* P*ssuA*
oWKS-1236	5′ - CAATTAAAACTCCCCCAATTCCC - 3′	**C. difficile* P*ssuA*
oWKS-1170	5′ - GGTTGGTGGAAGAAACATGG - 3′	**C. difficile* P*spoIIAA*
oWKS-1171	5′ - AATCCCTCCTTCAATAGTTTTG - 3′	**C. difficile* P*spoIIAA*
oWKS-1172	5′ - AAACGTGCGGATATTATAACTG - 3′	**C. difficile* P*spoIIE*
oWKS-1173	5′ - ACAACTGCTACACTTCTTTGC - 3′	**C. difficile* P*spoIIE*
oWKS-1174	5′ - ACTTCTACCTAGATATATTGGATTC - 3′	**C. difficile* P*spoIIGA*
oWKS-1175	5′ - ACTCAATATACACCATGTATCC - 3′	**C. difficile* P*spoIIGA*
oWKS-1168	5′ - GGATATCCCTAGTTGTTCATAG - 3′	**C. difficile* P*spoVG*

oWKS-1169	5′ - TAGCCCATTATGGCCTTC - 3′	**C. difficile* P*spoVG*
oWSK-1216	5′ - TCTCCTTATCTAATAGAAGAGTC - 3′	**C. difficile* P*tcdA*
oWKS-1217	5′ - ATTAAAGACATAAAAACCTCCTAG - 3′	**C. difficile* P*tcdA*
oWKS-1214	5′ - AATCAATAACTTAATCTAAGAATATC - 3′	**C. difficile* P*tcdB*
oWKS-1215	5′ - TAACTGTTTTCTATTAACTAAACTC - 3′	**C. difficile* P*tcdB*
oWKS-1212	5′ - TTATTGCTAAAATACTTTATTTATTAG - 3′	**C. difficile* P*tcdR*
oWKS-1213	5′ - CTAAAACAATTAATTCATAAAAAGAC - 3′	**C. difficile* P*tcdR*
oWKS-1330	5′ - CATAATATAGTTTTATACAAATAAAATAC - 3′	**C. difficile* P*tcdC*
oWKS-1331	5′ - AAATTCGTTACCATCATTTTTTTTAGAAAAC - 3′	**C. difficile* P*tcdC*
oWKS-1332	5′ - TTAGATGAAAAGAGATATTATTTTACAGATG - 3′	**C. difficile* P*tcdE*
oWKS-1333	5′ - GTGAACTACTGTGCATTCATCATAGTC - 3′	**C. difficile* P*tcdE*

*** Cycling conditions for EMSA probes**

All PCR reactions were performed using GoTaq polymerase (Promega) according to the manufacturer's instructions. Annealing and extension time for all PCR reactions was 30″. Annealing temperature was programmed according to the table below. After cycling, a final extension step of 7′ at 72°C was performed followed by a hold at 16°C until the products were purified on a column. Purified PCR products were stored at -20°C.

**Fragment (bp)**	**Annealing temperature**
B. subtilis P*abrB* (219)	55°C
B. subtilis P*abrB*-C4A (219)	55°C
B. subtilis P*abrB*-G2A (219)	55°C
B. subtilis P*abrB*-G2A-C4A (219)	55°C
B. subtilis P*citG* (287)	58°C
C. difficile P*spo0A* (289)	53°C
C. difficile P*sigH* (281)	55°C
C. difficile P*lplA* (184)	57°C
C. difficile P*ssuA* (277)	57°C

C. difficile P*spoIIAA* (197)	50°C
C. difficile P*spoIIE* (250)	50°C
C. difficile P*spoIIGA* (216)	50°C
C. difficile P*spoVG* (245)	50°C
C. difficile P*tcdA* (260)	57°C
C. difficile P*tcdB* (283)	57°C
C. difficile P*tcdR* (226)	50°C
C. difficile P*tcdC* (292)	50°C
C. difficile P*tcdE* (257)	52°C
